# Elevated Plasma IL-38 Concentrations in Patients with Acute ST-Segment Elevation Myocardial Infarction and Their Dynamics after Reperfusion Treatment

**DOI:** 10.1155/2015/490120

**Published:** 2015-12-24

**Authors:** Yucheng Zhong, Kunwu Yu, Xiang Wang, Xiaoya Wang, Qingwei Ji, Qiutang Zeng

**Affiliations:** ^1^Laboratory of Cardiovascular Immunology, Institute of Cardiology, Union Hospital, Tongji Medical College, Huazhong University of Science and Technology, Wuhan 430022, China; ^2^Department of Cardiology, Beijing Anzhen Hospital, Capital Medical University, Beijing 100029, China

## Abstract

*Objective.* Recent studies suggest that IL-38 is associated with autoimmune diseases. Furthermore, IL-38 is expressed in human atheromatous plaque. However, the plasma levels of IL-38 in patients with ST-segment elevation myocardial infarction (STEMI) have not yet to be investigated.* Methods.* On admission, at 24 h, at 48 h, and at 7 days, plasma IL-38, C-reactive protein (CRP), cardiac troponin I (cTNI), and N-terminal of the prohormone brain natriuretic peptide (NT-proBNP) levels were measured and IL-38 gene in peripheral blood mononuclear cells (PBMCs) was detected in STEMI patients.* Results.* The results showed that plasma IL-38 levels and IL-38 gene expression in PBMCs were significantly increased in STEMI patients compared with control group and were time dependent, peaked at 24 h. In addition, plasma IL-38 levels were dramatically reduced in patients with reperfusion treatment compared with control group. Similar results were also demonstrated with CRP, cTNI, and NT-proBNP levels. Furthermore, IL-38 levels were found to be positively correlated with CRP, cTNI, and NT-proBNP and be weakly negatively correlated with left ventricular ejection fraction (LVEF) in STEMI patients.* Conclusions.* The results indicate that circulating IL-38 is a potentially novel biomarker for patients with STEMI and IL-38 might be a new target for MI study.

## 1. Introduction

Acute myocardial infarction (AMI) still remains a leading cause of death worldwide. Following AMI, inflammatory mediators are released by the myocardium as a response to tissue injury and contribute to tissue repair and adaptive responses [1–4]. Circulating inflammatory markers, such as interleukin- (IL-) 6 and C-reaction protein (CRP), have been associated with adverse clinical outcomes, whereas elevations of the anti-inflammatory cytokine IL-10 have been associated with a more favorable prognosis [5–7]. Both mechanical and pharmacological reperfusion treatments, used to improve outcome in patients with acute myocardial infarction, might influence the inflammatory responses [8–10].

Interleukin-38 (IL-38) is a recently found receptor antagonist in the IL-1 ligand family and shares 43% homology with IL-36Ra and 41% homology with IL-1Ra [11]. IL-38 is predominantly expressed in the skin and in proliferating B-cells of the tonsil [12]. IL-38 binds to IL-36R similar to IL-36Ra, which can influence the proinflammation function of IL-36 and suppress* Candida*-induced IL-22 and IL-17 in a nonclassical dose-response method [13]. Moreover, polymorphisms in IL-38 were associated with CRP concentrations in humans [14] and were found to be significantly associated with coronary artery disease (CAD) [15]. In addition, IL-38 mRNA was found in human atheromatous plaques of coronary artery disease patients [16]. These results suggest an important role for this cytokine in human cardiovascular disease. However, the concentration and gene expression of IL-38 in peripheral blood were not investigated.

In ST-segment elevation myocardial infarction (STEMI), primary percutaneous coronary intervention (PCI) is the preferred treatment if it can be delivered within 2 hours from first medical contact. However, if primary PCI cannot be performed within the recommended time limits, thrombolytic therapy is the treatment of choice [17]. Previous studies demonstrated that the type of reperfusion treatment decided the predictive value of basal C-reactive protein levels for myocardial salvage in patients with AMI [18] and drug-eluting stents showed significantly lower plasma CRP levels after PCI compared with bare metal stent [19]. Nevertheless, whether the treatments affect IL-38 levels or not and the role of IL-38 in AMI is unknown.

In the present study, we measured the levels of plasma IL-38, C-reactive protein (CRP), cardiac troponin I (cTNI), and N-terminal of the prohormone brain natriuretic peptide (NT-proBNP) in STEMI patients with different treatments upon arrival into the emergency unit (on admission), 24 h, 48 h, and 7 days after admission and analyzed the correlation between IL-38 and the other parameters, including left ventricular ejection fraction (LVEF).

## 2. Methods

### 2.1. Patients

We recruited 102 patients who underwent diagnostic coronary angiography between June 2013 and February 2015 in the Union Hospital of Huazhong University of Science and Technology, Wuhan, China. Written informed consent was obtained from each patient. The study was approved by the Human Ethics Committee of Union Hospital of Huazhong University of Science and Technology. Patients contained 4 groups:

(1) Control group: chest pain syndrome (CPS) group (19 men and 7 women, mean age 55.40 ± 6.54) whose chest pain was not accompanied by ECG changes, coronary stenosis, or coronary spasm when an intracoronary injection of acetylcholine was given during coronary angiography.

STEMI patients (52 men and 24 women, mean age 57.28 ± 15.68) were divided into three groups according to therapy. Inclusion criteria were myocardial infarction confirmed by significant rise of cTNI and creatinine kinase MB (CK-MB) levels, ST-segment changes, and durative chest pain >30 mins, on admission within 12 h.

(2) Emergency PCI (24 men and 8 women, mean age 54.3 ± 8.81) group that were performed on with PCI within 12 h and did not include patients with failed thrombolysis.

(3) Thrombolysis (12 men and 6 women, mean age 44 ± 8.67) group performed on with thrombolytic treatment within 12 h, whose chest pain subsided and ST-segment dropped.

(4) Elective PCI (16 men and 10 women, mean age 57.9 ± 9.82) group performed on without thrombolytic treatment or PCI within two weeks.

Exclusion criteria were (1) patients treated with anti-inflammatory drugs such as nonsteroidal anti-inflammatory drugs and steroids; (2) suffering from acute or chronic infectious diseases and autoimmune diseases; (3) severe disorders of blood pressure, dysglycemia; (4) severe heart failure; (5) cardiomyopathy and heart valve disease; (6) liver disease, renal insufficiency, gastrointestinal diseases, and diseases of the blood system; (7) thyroid dysfunction and tumor; (8) surgery or trauma; and (9) a history of myocardial infarction and PCI.

### 2.2. Blood Samples

In the Control and STEMI group, blood samples were obtained from the patients upon arrival into the emergency unit (on admission), 24 h, 48 h, and 7 days after admission. The samples were collected into sodium heparin Vacutainers (Becton-Dickinson). The PBMCs were prepared by the Ficoll density gradient for analysis by real-time polymerase chain reaction (PCR). Blood was centrifuged for 10 min at 2000 ×g and plasma was stored at −80°C until further use.

### 2.3. ELISA

The levels of plasma IL-38 (Adipogen AG, Liestal, Switzerland) and CRP (Biocalvin) were measured by an enzyme-linked immunosorbent assay (ELISA), following the manufacturer's instructions. The other parameters were from the Biochemical Laboratory, Institute of Cardiology, Union Hospital. The minimal detectable concentration was 40 pg/mL for IL-38. The ELISA intra-assay and interassay coefficients of variation were <5% and <10%, respectively. All of the samples were measured in duplicate.

### 2.4. Real-Time PCR

Total RNA was isolated from fresh PBMCs using an RNeasy kit. cDNA was synthesized using random hexamer primers and RNase H-reverse transcriptase (Invitrogen, USA). Relative quantitative real-time PCR was performed using SYBR Green I Premix ExTaq on the ABI Prism 7900 (Applied Biosystems, Foster, CA) following the manufacturer's instructions. The specific primers were as follows: IL-38 (5-AGGACCAGACACCAC-TGATTG-3 and 5-TGGGGGCACAAGGCTAAAAC-3). The quality of cDNA subjected to the RT-PCR was controlled by amplification of transcripts of *β*-actin. *β*-actin was analyzed using the following primers: 5-CCTAAGGCCAACCGTGAAAAG-3 and 5-TCTTCATGGTGCTAGGA-GCCA-3 [12]. Quantitative PCR was performed on ABI PRISM 7900 Sequence Detector system (Applied Biosystems) using SYBR Green I Assay (Takara Biotechnology). Relative gene expression level (the amount of target, normalized to endogenous control gene) was calculated using the comparative Ct method formula 2^−ΔΔCt^  [20].

### 2.5. Doppler Echocardiography

Patients underwent M-mode and 2D-echocardiography using a GE ViVidE7 ultrasonography machine (GE Healthcare, America) with a transthoracic 1.5–4.3 MHz probe (M5S-D). Left ventricular end-diastolic diameter (LVEDD) and fractional shortening were measured. LVEF was calculated from apical four-chamber position by the area-length method.

### 2.6. The Gensini Score

The severity of coronary stenosis in patients was estimated by the Gensini coronary score following coronary angiography. The Gensini score was computed by assigning a severity score to each coronary stenosis according to the degree of luminal narrowing and its geographic importance. Reduction in the lumen diameter and the roentgenographic appearance of concentric lesions and eccentric plaques were evaluated (reductions of 25, 50, 75, 90, and 99% and complete occlusion were assigned Gensini scores of 1, 2, 4, 8, 16, and 32, resp.). The score was then multiplied by a factor that incorporates the importance of the lesion's position in the coronary arterial tree as follows: 5 for the left main coronary artery; 2.5 for the proximal left anterior descending coronary artery (LAD) or left circumflex artery (LCX); 1.5 for the mid-LAD; and 1 for the distal LAD, the right coronary artery, or the mid-distal LCX.

### 2.7. Statistical Analysis

The results are expressed as the mean ± SD unless otherwise indicated. The data of IL-38 and NT-proBNP are skewed distribution, and the statistical analysis is performed after ln transformation. Comparisons between the 2 groups were performed using Student's *t*-test. One-way ANOVA was used for multiple comparisons between ≥3 groups, followed by the LSD test. Spearman's correlation was used to calculate the correlations between the plasma biomarker levels and the other measured parameters. The Chi-Square test was used in comparison of the qualitative data. GraphPad Prism 6.0 was used for the statistical analysis. The significance level was set at *P* < 0.05.

## 3. Results

### 3.1. Baseline Characteristics

Baseline clinical characteristics between the control and patients with STEMI are presented in Table 1. Mean age and gender disturbance were balanced between the groups. There were no significant differences in weight index, vital signs (blood pressure, pulse rate, and temperature), HbA1c, history of diseases, or tobacco use among the four groups. The Gensini score was significantly higher in patients with STEMI than in patients with chest pain syndrome. Conversely, the LVEF was lower in patients with STEMI than in patients with chest pain syndrome. The other parameters of each group, including lipid and lipoprotein fractions, and prehospital medications are listed in Table 1.

### 3.2. The Expression of IL-38 in Periphery Blood of STEMI Was Increased

**Figure 1:**
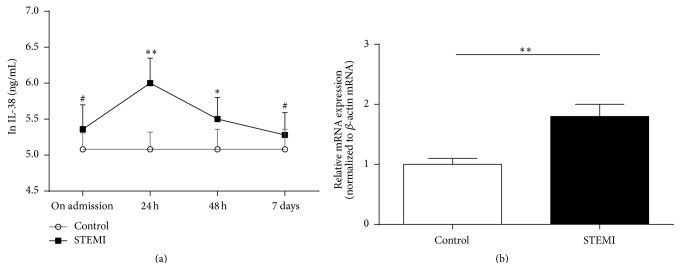
The IL-38 protein in plasma and IL-38 gene in PBMCs of STEMI patients. (a) Plasma IL-38 from STEMI patients. IL-38 protein was qualified by ELISA kit at different time-point. The statistical analysis is performed after ln transformation. (b) The IL-38 gene in PBMCs of STEMI patients. IL-38 gene expression was detected with RT-PCR at 24 h. STEMI: ST-segment elevation myocardial infarction; Control group: chest pain syndrome. Data are presented as mean ± SD. ^#^ *P* > 0.05, ^*∗*^ *P* < 0.05, and ^*∗∗*^ *P* < 0.01 versus Control.

IL-38 was found to be expressed in the heart, placenta, fetal liver, spleen, thymus, and tonsil [11, 12]. However, nobody detected its expression in blood and its relationship with STEMI. Here, we measure IL-38 in plasma using ELISA and in PBMCs using RT-PCR of 76 STEMI and 26 control patients. As shown in Figure 1(a), plasma IL-38 in patients with STEMI was slightly increased compared with those in patients with chest pain syndrome on admission, although there was no significant difference. The IL-38 concentrations in patients with STEMI peaked at 24 h and were significantly increased compared with those in patients with chest pain syndrome. Subsequently, the IL-38 concentrations were decreased quickly at 48 h and its levels at 7 days were almost the same as those on admission. In addition, there was no significant difference in plasma IL-38 levels between patients with STEMI and patients with chest pain syndrome at 7 days.

Above results indicated that the peak of IL-38 expression was 24 h; thus, its expression in PBMC was also investigated. As shown in Figure 1(b), RT-PCR showed that there was 1.8-fold increase of IL-38 gene in patients with STEMI compared with those in patients with chest pain syndrome at 24 h.

### 3.3. The Plasma IL-38 Concentrations and Other Measured Parameters after Different Reperfusion Strategies

To investigate the change of IL-38 production in STEMI patients after different reperfusion strategies, the STEMI patients were divided into Emergency PCI group, Thrombolysis group, and Elective PCI group. Then, we detected the plasma IL-38 levels on admission, at 24 h, at 48 h, and at 7 days and compared them between these three groups and the Control group. At the same time, CRP, cTNI, and NT-proBNP were examined.

**Figure 2:**
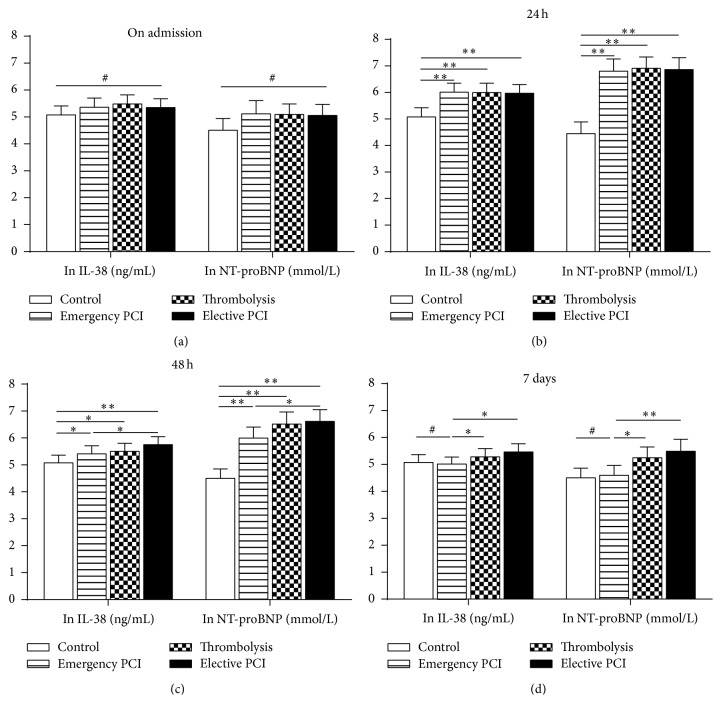
Plasma IL-38 and NT-proBNP from STEMI patients with different reperfusion strategies. IL-38 and NT-proBNP were qualified by ELISA kit in four groups (Control, Emergency PCI, Thrombolysis, and Elective PCI) at different time-points (on admission, 24 h, 48 h, and 7 days). The statistical analysis is performed after ln transformation. Data are presented as mean ± SD. ^#^ *P* > 0.05, ^*∗*^ *P* < 0.05, and ^*∗∗*^ *P* < 0.01.

**Figure 3:**
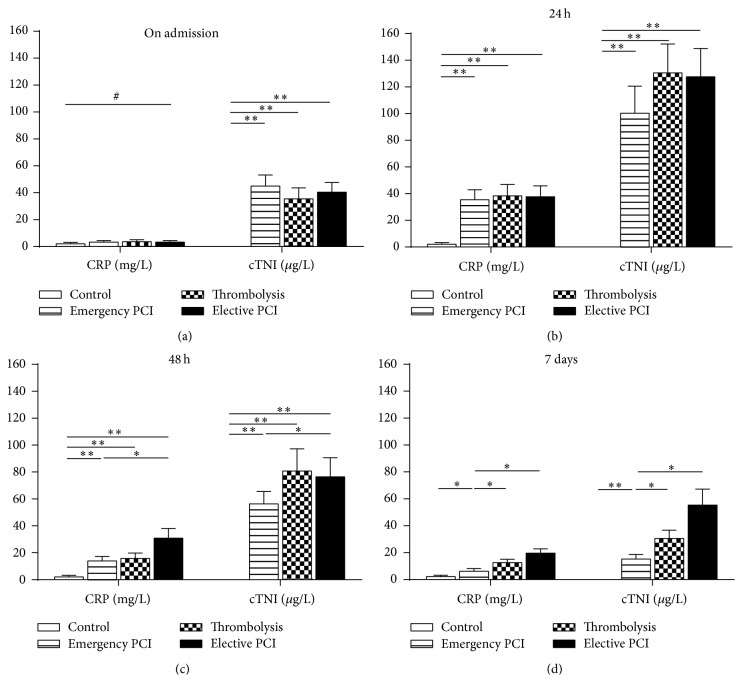
Plasma CRP and cTNI from STEMI patients with different reperfusion strategies. CRP and cTNI were qualified by ELISA kit in four groups (Control, Emergency PCI, Thrombolysis, and Elective PCI) at different time-points (on admission, 24 h, 48 h, and 7 days). Data are presented as mean ± SD. ^#^ *P* > 0.05, ^*∗*^ *P* < 0.05, and ^*∗∗*^ *P* < 0.01.

On admission, for IL-38, CRP, or NT-proBNP levels, there was no significant difference among these four groups (Figures 2(a) and 3(a)). Nevertheless, the cTNI concentrations were significantly increased in the STEMI patients compared with those in control patients (Figure 3(a)).

At 24 h, all parameters including IL-38 peaked in the STEMI patients but not in control patients. In addition, for all parameters, no differences were found among Emergency PCI group, Thrombolysis group, and Elective PCI group (Figures 2(b) and 3(b)).

At 48 h, all measured parameters started to decline in plasma of Emergency PCI group, Thrombolysis group, and Elective PCI group. However, the falling range was different among different treatments. For IL-38 or CRP levels, there was a more significant decline in Emergency PCI group or Thrombolysis group than that in Elective PCI group. In addition, for NT-proBNP or cTNI levels, there was a more significant decline in Emergency PCI group than that in Elective PCI group or Thrombolysis group (Figures 2(c) and 3(c)).

At 7 days, the IL-38 or CRP levels almost returned to normal. However, for all measured parameters, there was a more significant decline in Emergency PCI group than those in Elective PCI group or Thrombolysis group (Figures 2(d) and 3(d)).

### 3.4. Relationship between Plasma IL-38 Concentrations and Other Measured Parameters

**Figure 4:**
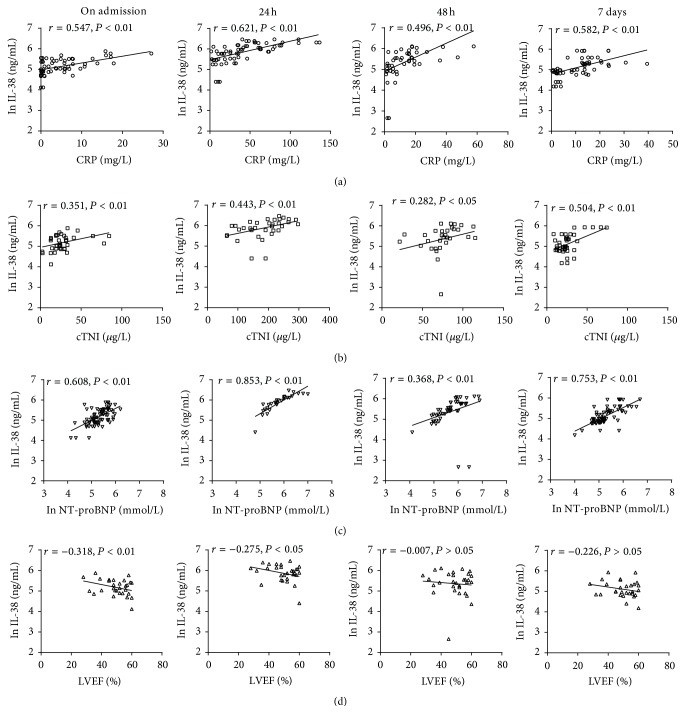
Spearman's correlation between IL-38 and CRP, cTNI, NT-proBNP, and LVEF in patients with STEMI. (a)–(c) IL-38 levels were positively correlated with CRP, cTNI, and NT-proBNP in STEMI patients at each time-point. (d) IL-38 concentrations were weakly negatively correlated with LVEF in STEMI patients at each time-point. Data are presented as mean ± SD.

We assessed whether the plasma IL-38 levels were associated with the Gensini score used to quantify the severity of coronary artery stenosis in CAD. There was no significant correlation between IL-38 and the Gensini score (data not shown). We further assessed whether IL-38 levels were associated with CRP, cTNI, NT-proBNP, and LVEF in patients with STEMI. The results showed that higher IL-38 levels were positively correlated with CRP, cTNI, and NT-proBNP in STEMI patients at each time-point (*P* < 0.01) (Figures 4(a), 4(b), and 4(c)) but not with other parameters (data not shown), whereas IL-38 levels were weakly negatively correlated with LVEF in STEMI patients on admission (*P* < 0.01) or at 24 h (*P* < 0.05) but not at 48 h or at 7 days (*P* > 0.05) (Figure 4(d)).

## 4. Discussion

In this study, plasma IL-38, CRP, cTNI, and NT-proBNP and IL-38 gene expression in PBMCs were investigated in STEMI patients at four time-points (on admission, 24 h, 48 h, and 7 days). The results showed that the expression of IL-38 in periphery blood of STEMI was increased, while plasma CRP, cTNI, and NT-proBNP levels were significantly increased in patients with STEMI compared with chest pain syndrome patients too. In addition, we found that the reperfusion strategies could decrease the above parameters, and IL-38 levels were positively correlated with CRP, cTNI, and NT-proBNP but were weakly negatively correlated with LVEF.

STEMI is usually associated with inflammation and develops into severe complications. During AMI, several mediators (including cytokines) interact, resulting in a net effect on myocardial remodeling [21]. Anti-inflammation strategy may be good for myocardial prognosis, but some anti-inflammatory cytokines such as IL-10 and TGF-*β* reduced in acute coronary syndrome (ACS) patients, reflecting the imbalance in systemic cytokine response following an ACS [22–24]. IL-38 is already proved as an anti-inflammatory cytokine [13] and found to be elevated in plasma of STEMI patients in our study, indicating that IL-38 might be induced from some kinds of activated cells. IL-38 is reported to be predominantly expressed in the skin and in proliferating B-cells of the tonsil [25], and we found that it increased in PBMCs of STEMI patients, indicating that leukocytes were important sources of plasma IL-38. Indeed, leukocyte is an important inflammatory cell in STEMI patients and is involved in myocardial necrosis and repair after STEMI [26]. Circulating PBMCs could express high level of proinflammatory cytokines, TNF-alpha, and IL-6, which were also increased in plasma [27]. For the anti-inflammatory cytokine, our previous study demonstrated that another IL-1 family member, IL-37, was significantly increased in ACS patients compared to control patients [28]. Recently, van de Veerdonk et al. found that similar to IL-36Ra, IL-38 could inhibit the production of IL-8, IL-17, and IL-22 in human PBMCs [13]. These results suggested there was a balance of inflammation and anti-inflammation in ACS patients. In addition, our unpublished data suggested that overexpression of IL-37 could inhibit myocardial remodeling after AMI in mice. Thus, we speculated that, as an anti-inflammatory cytokine, elevated IL-38 might antagonize an inflammation response in STEMI patients. However, whether elevated plasma IL-38 was derived from injured heart or arteries besides leukocytes deserves to be investigated and the exact role of IL-38 should be verified in animals.

Nevertheless, we found that IL-38 was decreased in patients after reperfusion strategies in our study at 48 h or 7 days, especially after emergency PCI treatment. After AMI, myocardial necrosis triggers a cytokine cascade. If reperfusion of the infarcted area is initiated, it is attended by an intense inflammatory reaction. Despite this potential injury in a short time, substantial evidence suggests that reperfusion enhances cardiac repair improving patient survival [3]. The faster fall in the levels of IL-38 after reperfusion strategies in our study indicated a decreased inflammatory reaction at 48 h or 7 days. We guessed that this was attributed to a reduced infarcted area and drug-eluting stents. Indeed, it was reported that drug-eluting stents showed significantly lower plasma CRP levels after emergency PCI compared with bare metal stents [19], which was observed at 7 days but not at 24 h and was consistent with our study.

Next, the results revealed that IL-38 levels were positively correlated with the traditional markers (cTNI, NT-proBNP, and CRP) and were weakly negatively correlated with LVEF. Cardiac troponin I (cTNI) has been extensively studied as a diagnostic and prognostic marker in ACS, and an increase in cTNI circulating levels is highly indicative of myocardium injury [29]. Moreover, previous studies showed that cTNI but not cardiac troponin T (cTNT) induces severe autoimmune inflammation in the myocardium [30]. These results indicate that cTNI could not only stand for the extent of cardiac injury but also predict autoimmune inflammation after AMI. Because of positive correlation with cTNI levels, IL-38 levels might be an indicator of myocardium injury or autoimmune. Another marker, NT-proBNP, is released from the cardiac ventricles in response to increased wall stress [31] and rises rapidly over 24 h after STEMI [32, 33]. When measured two to seven days after infarction, elevated levels of NT-proBNP identified patients with lower survival and were independent predictors of poor outcome [34]. We found that NT-proBNP fall faster after emergency PCI or thrombolysis than the Control group. CRP is one of the acute phase reactants, mainly produced by the liver, and recent studies have shown that elevated serum levels of CRP have been widely considered to be nonspecific but sensitive markers of the acute inflammatory response. A reduction in the rise of CRP has been shown to indicate reperfusion efficacy and a patent infarct-related coronary artery [10, 35]. A high CRP after AMI predicts infarct expansion, cardiac rupture, and mortality [5, 10, 36]. Infarcted tissue can also raise CRP. Thus, it is also possible that a reduction in myocardial infarct size and inflammation reaction by emergency PCI or thrombolysis is responsible for the reduction in CRP in this group. The positive correlation between IL-38 levels and CRP levels demonstrates that IL-38 might be a predictor of infarct expansion, cardiac rupture, and mortality. In contrast, correlation analyses also demonstrated that increased IL-38 levels were weakly negatively correlated with LVEF only on admission and at 24 h.

Approximately a half of the study population is diabetic in STEMI patients. Actually, we analyzed the levels of IL-38 in STEMI patients and found no significant difference between patients with diabetes and without diabetes (data not shown). Furthermore, there was no significant difference in HbA1c between STEMI patients and control patients and between STEMI patients with diabetes and without diabetes, indicating good blood sugar control during the past six months and reasonable explanation for unchanged IL-38 in STEMI patients with diabetes compared to those without diabetes. Previously, the therapeutic agents that contained biguanides and sulfonylurea were used to control diabetes and antagonize the chronic inflammation. Thus, AMI, an acute coronary event, dominates inflammation in these patients. However, the exact role of IL-38 on diabetes remains to be investigated.

Based on the above finding, plasma IL-38 levels could be a rule for judging whether PCI was successful or not and stand for the extent of antagonizing inflammation reaction in body. However, whether its significance was different from other inflammatory markers already available deserves to be investigated. On the one hand, as 7 days of follow-up was not enough, the exact relation between IL-38 and cardiac remodeling or clinic outcome needs to be studied further, and 1 month or 1 year may be needed. On the other hand, the mechanism and exact role of IL-38 on MI should be verified in animal model.

*Study Limitation.* This study has several limitations. First, sample size is not too big in our study. It is difficult that patients stay in hospital for more than 7 days. Second, there was a lack of heart samples to directly link circulating and tissue concentrations of IL-38 in our study. Ideally, correlation should have been established between local and systemic inflammatory or anti-inflammatory marker. Third, unlike other markers, the differences in IL-38 between different times and between different groups are minimal, indicating that the use of IL-38 as a diagnostic and prognostic marker of damage is uncertain.

## 5. Conclusion

In conclusion, the relationship between plasma IL-38 concentrations and CRP, cTNI, NT-proBNP, or LVEF indicates that IL-38 might be a predictor of successful reperfusion and a diagnostic and prognostic marker in STEMI. These issues need to be further investigated in animal experiments and in larger studies with a long time of follow-up.

## Figures and Tables

**Table 1:** Clinical characteristics of patients.

Characteristics	STEMI (*n* = 76)	Emergency PCI (*n* = 32)	Thrombolysis (*n* = 18)	Elective PCI (*n* = 26)	Control (*n* = 26)
Male *n* (%)	52 (68.4)	24 (75.0)	12 (66.7)	16 (61.5)	19 (73.1)
Age (years)	57.2 ± 15.6	54.3 ± 8.8	44.0 ± 8.6^*∗*^	57.9 ± 9.8	55.4 ± 6.5
Weight index	24.2 ± 0.8	24.4 ± 1.0	24.7 ± 0.9	23.7 ± 0.5	24.9 ± 0.7
SBP	128.5 ± 16.8	134.2 ± 13.6	128.3 ± 10.4	119.2 ± 14.3	124.1 ± 12.5
DBP	74.5 ± 5.6	72.4 ± 9.7	80.4 ± 9.3	69.9 ± 8.1	78.0 ± 6.7
Pulse rate	76.9 ± 3.5	77.2 ± 1.7	76.2 ± 2.4	77.2 ± 1.9	75.7 ± 2.2
Temperature	36.4 ± 0.06	36.2 ± 0.05	36.3 ± 0.08	36.4 ± 0.07	36.4 ± 0.04
LVEF	0.48 ± 0.04^*∗*^	0.46 ± 0.05^*∗*^	0.47 ± 0.02^*∗*^	0.49 ± 0.04^*∗*^	0.64 ± 0.04
TC (mmol/L)	4.88 ± 0.36^*∗*^	5.22 ± 0.17^*∗*^	4.45 ± 0.25	4.59 ± 0.23	3.93 ± 0.17
LDL-C (mmol/L)	2.87 ± 0.33	3.24 ± 0.15^*∗*^	2.71 ± 0.21	2.72 ± 0.18	2.54 ± 0.15
Creatinine (*µ*mol/L)	84.67 ± 12.65	96.18 ± 38.17	76.10 ± 11.33	82.27 ± 10.89	80.58 ± 13.23
Trioxypurine (*µ*mol/L)	360.35 ± 30.62	360.91 ± 29.13	340.80 ± 20.67	353.90 ± 34.61	309.2 ± 31.59
HbA1c (%)	5.98 ± 0.80	6.03 ± 0.83	5.89 ± 0.81	5.96 ± 0.75	5.56 ± 0.76
Gensini score	67.23 ± 36.43	65.60 ± 40.72	69.72 ± 42.51	70.30 ± 34.11	66.50 ± 31.62
*History, n, (%)*					
Smoking	60 (78.95)	26 (81.25)	16 (88.89)	18 (69.23)	13 (50.00)
Family history	48 (63.16)^*∗*^	22 (68.75)^*∗*^	10 (55.56)^*∗*^	16 (61.54)^*∗*^	5 (19.20)
Hypertension	53 (69.74)	25 (78.13)	14 (77.78)	14 (53.85)	13 (50.00)
Diabetes	40 (52.63)^*∗*^	20 (65.50)^*∗*^	9 (50.00)^*∗*^	11 (42.31)^*∗*^	3 (11.50)
Hyperlipidemia	38 (50.00)	18 (56.25)	8 (44.44)	12 (46.15)	15 (57.70)
*Medications*					
Nitrates	20 (26.32)	10 (31.25)	4 (33.33)	6 (23.08)	13 (50.00)
*β*-blockers	40 (52.63)	22 (68.75)	8 (44.44)	10 (38.50)	10 (38.50)
ACEI/ARB	36 (47.37)	15 (46.88)	12 (66.67)	9 (34.62)	14 (53.80)
Statins	32 (42.11)	11 (34.38)	9 (50.00)	12 (46.15)	19 (73.10)
Antiplatelet/anticoagulants	76 (100)	32 (100)	18 (100)	26 (100)	26 (100)

^*∗*^ *P* < 0.05 versus Control. SBP: systolic blood pressure; DBP: diastolic blood pressure; LVEF: left ventricular ejection fraction; TC: total cholesterol; LDL-C: low-density lipoprotein cholesterol; ACEI: angiotensin-converting enzyme inhibitors; ARB: angiotensin receptor blocker.

## Abbreviations

CAD: Coronary artery disease
PCI: Percutaneous coronary intervention
STEMI: ST-segment elevation myocardial infarction
CRP: C-reactive protein
PBMCs: Peripheral blood mononuclear cells
LVEF: Left ventricular ejection fraction
ACS: Acute coronary syndromes
cTNI: Cardiac troponin I
NT-proBNP: N-terminal of the prohormone brain natriuretic peptide
cTNT: Cardiac troponin T
CPS: Chest pain syndrome.
